# Expectations Regarding Dental Practice: A Cross-Sectional Survey of European Dental Students

**DOI:** 10.3390/ijerph17197296

**Published:** 2020-10-06

**Authors:** Thomas Gerhard Wolf, Ralf Friedrich Wagner, Oliver Zeyer, Duygu Ilhan, Tin Crnić, Ernst-Jürgen Otterbach, Guglielmo Campus

**Affiliations:** 1 Department of Restorative, Preventive and Pediatric Dentistry, School of Dental Medicine, University of Bern, CH-3010 Bern, Switzerland; rwagner@kzvnr.de (R.F.W.); guglielmo.campus@zmk.unibe.ch (G.C.); 2 Department of Periodontology and Operative Dentistry, University Medical Center of the Johannes Gutenberg-University Mainz, 55131 Mainz, Germany; 3 FVDZ Free Association of German Dentists, 53177 Bonn, Germany; e-j.otterbach@fvdz.de; 4 Association of Statutory Health Insurance Dentists North Rhine (KZV Nordrhein), 40237 Düsseldorf, Germany; 5 SSO Swiss Dental Association, 3000 Bern, Switzerland; oliver.zeyer@sso.ch; 6 Private Practice, Valikonağı Street, 34635 Istanbul, Turkey; duyguilhan@yahoo.com; 7 Turkish Dental Association (Türk Dişhekimleri Birliği), 06530 Çukurambar, Cankaya/Ankara, Turkey; 8 EDSA European Dental Students’ Association, D02 PN40 Dublin, Ireland; president@edsaweb.org; 9 Department of Medicine, Surgery and Experimental Sciences, School of Dentistry, University of Sassari, I-07100 Sassari, Italy

**Keywords:** future career, expectations, liberal dental practice, Europe, dental students

## Abstract

Obtaining information on expectations among dental students regarding their career planning was the main purpose of this observational online survey. The questionnaire was designed with 18 items in five different languages: English, French, German, Italian and Spanish. Data were collected on nationality, age, sex, country of residence, university attended, semester, expected year of graduation and expectations about future career. More than 3000 participants (*n* = 3851, 2863 females 74.34% and 988 males 25.66% with a sex ratio of 0.35) participated in the survey. Almost one-third (31.29%) of the participants plan to start their own practice at least three years after vocational training, a quarter (25.76%) after three, and only 12.59% after one year. A positive influence of the family in the decision to start a practice was observed in 50.07% of the sample with a statistically significant difference regarding sex (*p* < 0.01). Almost one-third of the participants did not wish to work in an institution run by private equity or insurance companies, while 21.79% would work in that environment (*p* < 0.01). European dental students desire mainly to become self-employed and start their own practice. New professional practices also offer them options for their future career that they have not yet decided on or thought about.

## 1. Introduction

New generations of dentists will face very different times and different problems but also new opportunities. Non-operative treatment rather than operative treatment [1], antimicrobial resistance [2], the management of sugar [3] and the impact of tobacco consumption on oral health [4] will be more and more relevant in the future, not to mention the fact that the current COVID-19 pandemic [5,6,7] will also have a significant impact on the future of the dentistry profession. Moreover, topics such as green dentistry, sustainability and recycling management to mitigate climate change, artificial intelligence and big data (e-health), and tele-dentistry [8] are changing dentistry worldwide. A wide range of employment opportunities from which dentistry graduates can choose directly after their studies are now available [9]. A general trend towards larger forms of practice such as group practices or dental centers can be observed in Europe as well as in North America [9,10,11,12]; practice networks and group practices, increasingly owned by private equity firms, are gaining ground, accompanied by changes in the provision of dental services [9,10].

It is imperative to gain reliable information in order to create the framework conditions for optimal patient care, both in rural and urban areas [13,14]. However, little information is available on current students’ choice of occupation or their desire to pursue a profession later [7,12]. Therefore, the University of [name anonymized], in cooperation with the [name anonymized], has set itself the goal of conducting a survey among dental students in geographical Europe (WHO Region Europe). We hypothesized that there are some related factors regarding the expectations of European dental students and their future career planning. The aim of this study was therefore to collect and provide data on how the future generation of dentists imagine work and how their expectations will shape their career planning.

## 2. Materials and Methods

The European Regional Organization (ERO) of the FDI World Dental Federation consists of member associations from 37 of 53 countries in the WHO European Region [15]. The region has a population of almost 900 million people [16]. In comparison, the population of the European Union (27 EU member states, after the United Kingdom’s exit from the EU) in 2020 will be around half of the WHO European Region, at 447,706,209 people (218,237,878 males and 228,586,686 females) [17]. The average density of dentists in the ERO countries is 1570 inhabitants per dentist; the 353 dental universities produce a total of 16,619 dental graduates annually [9].

### 2.1. Questionnaire

Dentistry students from a total of 40 nationalities that studied in the countries of the ERO-FDI (WHO Europe) region took part in the current study. The study was conducted by means of an online self-administered online questionnaire in five languages: English, French, German, Italian and Spanish. The dental students were contacted by the national dental associations of the respective countries and supporting associations: the European Regional Organization of the FDI World Dental Federation (Bern/Geneva, Switzerland), the International Association of Dental Students (IADS, Geneva, Switzerland), the Free Association of German Dentists (FVDZ, Bonn, Germany) and the European Dental Students’ Association (EDSA, Dublin, Ireland). The online form contained brief information stating the aim of the survey and was accessible via a link that was sent to the participants by e-mail. The online website contained a question tool allowing participants of the survey to fill in the questions with answers directly on computer, tablet or mobile devices (https://www.survey.consulimus.de/english/umfragen/dental-students-in-europe.html). All information was provided voluntarily and was evaluated anonymously.

All procedures were in accordance with the ethical standards of the local research committee and with the 1964 Helsinki declaration and its later amendments, or with comparable ethical standards. Swiss law on human research (Humanforschungsgesetz, HFG) does not require ethics committee approval to collect and analyze anonymous data. Informed consent was obtained from all study participants by accessing the online survey. The questionnaire was developed on the basis of similar questionnaires from the literature [7,18,19,20,21]. Information was requested about nationality, age, sex, country of residence, university, semester, expected year of graduation, mandatory postgraduate (vocational) training after graduation, initial career plans after vocational training, the ideal practice type and reasons therefore, the intended time to work as self-employed, family-life influence on the career, reasons for working as an employed dentist and settings of an employed dentist, type of practice and sex ratio at the university (semester/year). 

### 2.2. Participating Countries

The countries were combined as follows to perform the statistical calculation: Balkans: Bosnia/Herzegovina, Croatia (EU), Montenegro; Baltic/Scandinavia: Denmark, Finland, Latvia, Lithuania, Norway, Sweden; Middle East: Afghanistan, Iran, Iraq, Israel, Lebanon, Palestine, Syria, United Arab Emirates (UAE); Non-EU: Azerbaijan, Kazakhstan, Russia, Ukraine, United Kingdom, United States of America; Other EU: Austria, Belgium, Bulgaria, Cyprus, Czech Republic, France, Greece, Hungary, Ireland, Luxembourg, Malta, Netherlands, Poland, Portugal, Romania, Slovakia, Slovenia, Germany, Italy, Spain, Switzerland and Turkey. The questionnaire was tested by the ERO-FDI Working Group members and by several dental students that were invited to the Working Group meetings before being sent online.

### 2.3. Data Analysis

Answers to the questionnaire were inserted in Excel^TM^ (Microsoft Corp., Redmont, WA, USA) 2019 for Mac. The data were cleaned and then transferred to STATA16^TM^ (StataCorp LLC, College Station, TX, USA) for their statistical analysis. Data sets with fewer than 5 answered questions (<25%) were removed from the final data set and not used for the survey (*n* = 129). Absolute and relative frequencies were calculated for each item. Difference in proportion was evaluated with the χ^2^ test or the Fisher exact test if one cell had a value of less than five. Multiple testing for *post hoc* estimation was calculated, such as the number of observed frequencies, expected frequencies, percentage, and contribution to the chi-square. Some items were constructed using the Likert scale [22]. A multinomial logistic regression model was run, using as dependent variables planning to open one’s own practice by sex, nationality, family and main reason to open the practice. The significance level was set at *p* < 0.05.

## 3. Results

**Figure 1:**
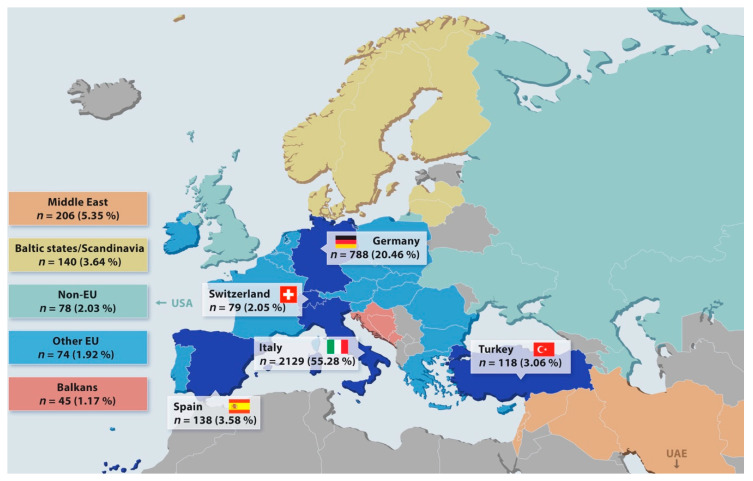
Map of Europe with information on the nationality of study participants.

A total sample of 3851 subjects (2863 females and 988 males) filled in the questionnaire, with a male/female ratio of 0.34. The no-response rate was 3.50% (*n* = 129). A map of Europe with information on the nationality of study participants is shown in Figure 1.

### 3.1. Sample Distribution

The distribution of the sample across nationality and sex was statistically significantly different according to nationality (χ^2^_(10)_ = 20.82 *p* < 0.01), the majority of participants were Italians (55.28%) and Germans (20.46%) (Table 1).

### 3.2. Influence of Sex on Owning a Practice

The proportion of female participants, as self-reported by the participants, was mainly between 40% and 70% for all semesters (data not in table). Sex and planning period for owning a practice was statistically significantly associated (χ^2^_(3)_ = 44.63 *p* < 0.01); the majority of the participants (31.29%) would like to decide to have their own practice at least three years after the period of assistance (vocational training), with the proportion of women outweighing that of men (Table 2).

### 3.3. Association between Sex and Family Influence on the Decision to Own a Practice

The same figures were presented at three years (25.76%), two years (12.67%) and one year (12.59%), with no statistically significant differences between sexes. The association between sex and the influence of family planning on the decision to start a practice was seen as positive by the vast majority (48.10%), women more often (29.16%) than men (18.94%). A neutral family influence was recorded by 12.88% of the sample, with similar results regarding sex. More than 39% of the sample reported the influence of family as negative (Table 3).

### 3.4. Influence of Sex on Agreement to Work in an Institution Run by Private Equity or Insurance Companies

The association between sex and agreement to work in an institution run by private equity or insurance companies (Table 4) was rejected by more than half (53.42%) of the sample (χ^2^_(3)_ =20.00 *p* < 0.01).

### 3.5. Students’ Planning to Work in a Private Equity or Insurance-Led Dental Center

Whether or not students plan to work in a dental center run by a private equity firm or insurance company in their later working life is a very heterogeneous question, but for many non-respondents the proportion of study participants who totally or partially disagreed (59.23%) outweighed those who were undecided (24.43%) or agreed (16.34%) (Table 5).

### 3.6. Planning to Open One’s Own Practice by Sex, Nationality, Family and Main Reason to Open the Practice

Table 6 displays the ordinal logistic regression for planning the establishment of one’s own practice by sex, nationality, family and main reason to open the practice.

Males were more open to establishing their own practice than women (OR = 0.75 _95%_CI = 0.60/0.94) and the main reason not to open was a financial issue (OR = 2.14 _95%_CI = 1.24/3.14).

## 4. Discussion

### 4.1. Change in Dental Profession

The transformation of the oral health sector is currently facing multiple challenges across Europe [23], and the adaptation of the dental profession to current trends must be followed in order to obtain information for designing and guiding oral health care [7,12,13,14]. In line with this need, an online survey was designed and carried out to obtain information about the wishes and expectations of dental students in Europe regarding their dental careers. The outcomes may prove highly relevant for developing sustainable oral health care in Europe.

### 4.2. Motivations for Studying Dentistry

Motivations for dental students deciding to study dentistry are most frequently the lack of admission to medical school, interest in dentistry, and the prestige and respect gained through the profession; a rarely mentioned reason is the influence of other dentists [24]. These findings were confirmed in an international European survey, where motivations such as independence, financial freedom, working with one’s hands, the ability to help people, but also status/prestige, time management and career diversity [25] were mentioned.

### 4.3. Sample Distribution

A higher number of female dentists was reported in the present survey, confirming a pattern present in many European countries [12,26]. Unfortunately, the distribution of participants in terms of nationality in the present study is uneven, with the highest percentage from Italy and Germany, probably due to poor access to students by national organizations and contact persons. Even with this limitation, the sample size is quite large. Another aspect that needs attention is that, depending on the semester, many students have not really considered the topic of a professional activity after graduation, resulting in a high number of non-responders.

### 4.4. Own Dental Practice

According to the present results, dental students would not like to establish their own practice until several years after their vocational training. While determining the reasons for this were not the aim of the current study, motivations for studying dentistry may have changed after the first years of gaining professional experience [27]. Students’ vision is challenged by changes affecting the healthcare system as well as their education. However, a long-term professional expectation is a favorable work-life balance and financial aspects, professional status, flexible work opportunities and job security are still perceived advantages of the dental profession [27]. This is also in line with the findings of the present study, that the main reasons for having one’s own practice are financial freedom and more flexibility in planning for a family. The fact that young dentists prefer private practice has been described [7,28], whereas the National Health Service in the UK also offers advantages for young colleagues such as a working environment with access to specialist training and, above all, clinical experience [27]. However, when considering employment and self-employment in one’s own/private practice, a distinction must be made depending on the country in which one lives. The different healthcare systems could have a direct influence on career choice, an issue that should be investigated further. Advantages of having one’s own practice could be job security, flexible work opportunities, independence of a career in health care, financial opportunities and social status [27,28,29,30,31]. A good balance between work life and personal life is a relevant desire as a long-term factor in students’ career prospects, whereas personal satisfaction is more relevant as a short-term factor [18], as are continuing professional education, financial stability and increasing knowledge and developing skills [29]. The number of self-employed individuals has decreased among health professionals [12,32]; while the income of the self-employed has decreased, the income of employees has remained constant.

### 4.5. Challenges in Being Self-Employed

Observing a trend towards the increasingly rapid growth of dental chains, facts such as high loan debt and less administrative work are reasons for young dentists to choose to be employed, a factor that was not examined in detail in this study, but will need further research. While in a Swiss study more than 65% of the participants said that they would like to work full-time in the future [7], the background knowledge that there is an increasing number of female dentists may have a significant impact on the professional practice of dentistry in different directions, with a possible increase in part-time employees due to family planning [33]. The main reasons cited by female dentists are care responsibilities and child-rearing [34,35]. This is interesting in that the information provided by the students in this study shows that the vast majority of them nevertheless regard the influence of family planning as neutral. With the increasing number of female dental professionals, a well-structured working environment and good time management could become more important in terms of personnel planning [25].

### 4.6. Liberal Dental Practice

An important factor in the dental profession, no matter what type of profession is chosen, is that this choice is made under the principles of liberal dental practice and ethical guidelines with a free choice of doctor and therapy and not primarily due to economic interests, state constraints and practice management that focuses primarily on financial rewards [12]. Although students often have little to no points of contact with such topics, it is clear that, as our study found, students are generally reluctant to work in a dental center run by a private equity firm or insurance company. In particular, it is important to consider the expectations and needs of the young dental professionals, while avoiding bottlenecks in dental care, which certainly needs to be taken into consideration. However, it has not yet been possible to confirm that rural and sparsely populated areas would expect an undersupply of dental services [14]. Sex differences will also be further examined. As part of the present trend towards more females in the dental profession, women may work 4–6 h less per week, have fewer patients and have their own practice less often than men [36]. However, in some European regions (e.g., the Swiss-German region), the number of women working in their own practice is increasing [11].

This study has some limitations. First, it is a cross-sectional study; we should pay attention to a temporal association. Second, there were no data on important factors for preparing detailed planning. Some students did not provide detailed information on owning a practice.

## 5. Conclusions

Some final considerations regarding the expectations of European dental students for their future career planning can be noted:

- Future dental professionals will be predominantly female, and the majority of future dentists would like to have their own practice in more than three years’ time.
- Moreover, dentistry students have different opinions about whether they want to work for an institution through a private equity or insurance company.

## Figures and Tables

**Table 1:** Distribution of the sample across nationality and sex.

Nationality	Sex Ratio (m/f) [12]	Sex Ratio (m/f)	Females	Males	Total	(%)
Balkans	0.54 (35:65)	0.41	32	13	45	1.17
Baltic/Scandinavia	0.25 (20:80)	0.71	82	58	140	3.64
Germany	0.54 (35:65)	0.46	538	250	788	20.46
Italy	1.13 (53:47)	0.57	1358	771	2129	55.28
Middle-East	0.67 (40:60)	1.54	81	125	206	5.35
Non-EU	0.67 (40:60)	0.73	45	33	78	2.03
Other EU	0.52 (35:65)	0.45	51	23	74	1.92
Spain	0.67 (40:60)	0.68	82	56	138	3.58
Switzerland	0.67 (40:60)	0.55	51	28	79	2.05
Turkey	1.22 (55:45)	0.57	75	43	118	3.06
Total			2395	1400	3795	98.55

Non-responders 56 χ^2^_(10)_ = 20.82 *p* < 0.01.

**Table 2:** Distribution of the sample across planning period for owning a practice and sex.

	Females *n* (%)	Males *n* (%)	Total *n* (%)
**One year**	254 (8.01)	211 (6.66)	485 (12.59)
**Two years**	242 (7.63)	246 (7.76)	488 (12.67)
**Three years**	519 (16.37)	423 (13.34)	992 (25.76)
**More than three years**	862 (27.19)	343 (10.82)	1205 (31.29)

Non-responders 681 χ^2^_(3)_ = 44.63 *p* < 0.01.

**Table 3:** Association between sex and family influence on the decision to own a practice.

	Females *n* (%)	Males *n* (%)	Total *n* (%)
**Positive**	1112 (29.16)	722 (18.94)	1834 (48.10)
**Negative**	742 (19.46)	746 (19.56)	1488 (39.02)
**Neutral**	255 (6.69)	236 (6.19)	491 (12.88)

Non-responders *n* 38 χ^2^_(3)_ = 42.55 *p* < 0.01.

**Table 4:** Association between sex and agreement to work in an institution run by private equity or insurance companies.

	Females *n* (%)	Males *n* (%)	Total *n* (%)
**Totally not agree**	540 (17.01)	434 (13.67)	974 (30.68)
**Not agree**	407 (12.82)	315 (9.92)	722 (22.74)
**Partially agree**	469 (14.77)	318 (10.02)	787 (24.79)
**Agree**	313 (9.86)	279 (8.79)	342 (10.77)
**Totally agree**	316 (9.95)	284 (8.94)	350 (11.02)

Non-responders *n* 676 χ^2^_(3)_ =20.00 *p* < 0.01.

**Table 5:** Association of students if they are planning to work in a private equity or insurance-led dental center.

Sex	Totally Disagree *n* (%)	Partially Diagree *n* (%)	Undecided *n* (%)	Partially Agree *n* (%)	Totally Agree *n* (%)	Total
**Females**	290 (24.68)	157 (13.36)	219 (18.64)	63 (5.62)	66 (5.62)	795 (67.66)
**Males**	184 (15.66)	65 (5.53)	68 (5.79)	29 (2.47)	34 (2.89)	380 (32.34)
**Total**	474 (40.34)	222 (18.89)	287 (24.43)	92 (7.83)	100 (8.51)	1175 (100)

Non-responders *n* 2696 χ^2^_(4)_ = 20.00 *p* < 0.01.

**Table 6:** Multilevel ordinal logistic regression about planning to open one’s own practice by sex, nationality, family and main reason to open the practice. The table includes the fixed-effect portion of our model, the estimated cut-points and the estimated variance components.

Variables	OR (SE)	*p*-Value	95% CI
**Sex (male)**	0.75 (0.09)	0.01	0.60–0.94
**Nationality (Italian)**	0.99 (0.13)	0.61	0.97–1.02
**Family opinion (positive)**	0.64 (0.10)	0.06	0.49–1.09
**Main reason (financial)**	2.14 (0.12)	0.03	1.24–3.14
**/cut1**	0.91 (0.90)	0.57	(0.65–1.26)
**/cut2**	3.24 (0.84)	<0.01	(2.32–4.53)
**/cut3**	10.88 (1.19)	<0.01	(7.64–15.33)
**Two years (Variable component)**	3.02 (0.20)		3.41–2.62
**Three years (Variable component)**	2.23 (0.19)		−1.89–2.60
**More than three years (Variable component)**	0.04 (0.17)		−0.29–0.37

Number of obs = 3299 Log likelihood = −1309.25 *p* < 0.01. Reference for dependent variable (one year).
